# Choosing Between Anti-TROP2 ADCs in Advanced Breast Cancer Without Head-to-Head Evidence: A Practical Evidence-Informed Decision Framework Endorsed by the Polish Society of Clinical Oncology

**DOI:** 10.3390/cancers18193227

**Published:** 2026-10-07

**Authors:** Piotr J. Wysocki, Katarzyna Pogoda, Ewa Wysocka, Barbara Radecka, Maciej Krzakowski

**Affiliations:** 1 Department of Oncology, Jagiellonian University—Medical College, 31-501 Krakow, Poland; 2 Department of Oncology, University Hospital, 30-688 Krakow, Poland; 3 Department of Breast Cancer and Reconstructive Surgery, Maria Sklodowska-Curie National Research Institute of Oncology, 02-781 Warsaw, Poland; katarzyna.pogoda@nio.gov.pl; 4 Scientific Students’ Society, Jagiellonian University—Medical College, 31-008 Krakow, Poland; ewa.1.wysocka@student.uj.edu.pl; 5 Department of Oncology, Institute of Medical Sciences, University of Opole, 45-052 Opole, Poland; 6 Department of Clinical Oncology, Tadeusz Koszarowski Cancer Center in Opole, 45-061 Opole, Poland; 7 Department of Lung and Chest Tumors, Maria Sklodowska-Curie National Research Institute of Oncology, 02-781 Warsaw, Poland; maciej.krzakowski@nio.gov.pl

**Keywords:** TROP2, antibody–drug conjugates, sacituzumab govitecan, datopotamab deruxtecan, advanced breast cancer, triple-negative breast cancer, HR-positive/HER2-negative breast cancer, treatment selection

## Abstract

Background/Objectives: Antibody–drug conjugates (ADCs) targeting trophoblast cell-surface antigen 2 (TROP2), including sacituzumab govitecan (SG) and datopotamab deruxtecan (Dato-DXd), have expanded treatment options for patients with advanced breast cancer. Although both agents target TROP2, they differ in molecular design, clinical evidence, toxicity profiles, and monitoring requirements. Without head-to-head trials, their relative clinical positioning remains uncertain. This review aimed to critically assess the available evidence and develop a practical framework to support individualized treatment selection between SG and Dato-DXd. Methods: This narrative critical review used a structured literature search of PubMed/MEDLINE and targeted searches of major oncology guidelines, regulatory documents, pivotal-trial publications, conference reports when full publications were unavailable, and relevant retrospective or translational studies. The evidence cutoff was 29 September 2026. Direct randomized-trial evidence, indirect clinical inference, and expert/practical considerations were explicitly distinguished. Results: SG and Dato-DXd have both demonstrated clinically meaningful activity in advanced breast cancer, but their evidence base, toxicity patterns, and practical requirements differ. Cross-trial differences in populations, treatment lines, comparator composition, follow-up, subsequent therapy, response assessment, and safety reporting preclude valid numerical comparisons of efficacy or toxicity between the two agents. SG is characterized predominantly by hematologic and gastrointestinal toxicity, whereas Dato-DXd is associated with stomatitis, ocular toxicity, and interstitial lung disease/pneumonitis. These distinctions, together with patient-specific comorbidities, prior toxicities, prior ADC exposure, logistical considerations, and local healthcare resources, may inform individualized treatment selection when both agents are clinically appropriate. Conclusions: In the absence of direct comparative trials, treatment selection between SG and Dato-DXd should not rely on cross-trial efficacy or safety comparisons. The proposed framework separates regulatory and guideline eligibility from drug-specific safety evidence, indirect clinical inference, patient preferences, and healthcare system considerations. It is intended as evidence-informed clinical guidance rather than a validated comparative treatment selection rule and requires prospective evaluation.

## Simple Summary

Two antibody–drug conjugates targeting TROP2, sacituzumab govitecan and datopotamab deruxtecan, are now available for the treatment of advanced breast cancer. Although both agents target the same protein, they differ in drug design, available evidence, toxicity profiles, and treatment-monitoring requirements. Because randomized trials have not directly compared them, choosing between these two drugs in clinical practice can be challenging. This review summarizes the available phase III evidence and current international recommendations and proposes a practical framework for treatment selection. The framework considers not only expected treatment efficacy and toxicity, but also patient comorbidities, monitoring requirements, and healthcare system resources. It is intended to support individualized clinical decision-making rather than provide a formal or validated treatment selection algorithm.

## 1. Introduction—The Growing Role of Antibody–Drug Conjugates in Oncology

Over the past decade, antibody–drug conjugates (ADCs) have become an important part of systemic therapy for breast cancer. By linking a tumor-directed monoclonal antibody to a cytotoxic payload, they allow more selective delivery of chemotherapy to cancer cells. Clinical development initially focused on HER2-directed agents such as trastuzumab emtansine and trastuzumab deruxtecan (T-DXd), but has since expanded to TROP2, which is broadly expressed across breast cancer subtypes.

Two TROP2-directed ADCs are now available in clinical practice: sacituzumab govitecan (SG) and datopotamab deruxtecan (Dato-DXd). As of September 2026, both have received authorization from the U.S. Food and Drug Administration (FDA) and the European Medicines Agency (EMA) in breast cancer, although the exact indications and supporting evidence differ across treatment settings and jurisdictions [1,2,3,4,5,6]. This creates a practical question for clinicians: how should we choose between two drugs that share the same target but differ in molecular design, efficacy data, toxicity, and monitoring requirements? This paper reviews the available evidence and proposes an evidence-informed framework for individualized treatment selection.

## 2. Methods

### 2.1. Review Design, Information Sources, and Evidence Selection

This article is a narrative critical review, not a systematic review or meta-analysis. A structured PubMed/MEDLINE search was supplemented by targeted review of FDA and EMA regulatory documents, NCCN, ESMO, AGO and ASCO guidance, ClinicalTrials.gov, and major oncology-congress reports, with an evidence cutoff of 29 September 2026. Search terms included the two agents, key phase III trials, breast cancer subtypes, toxicity, quality of life, biomarkers, and ADC sequencing. Priority was given to phase III randomized trials, peer-reviewed publications, current product labels, and guidelines. Conference abstracts were included only when relevant results were not yet available in full publication. Retrospective, real-world, pharmacogenetic, and preclinical studies were used only to address questions not covered by randomized evidence. Eligible evidence included clinical efficacy and safety, regulatory and guideline information, patient-reported outcomes, biomarkers, pharmacogenetics, sequencing, and translational data directly relevant to the framework. Duplicate reports, non-breast-cancer studies without direct relevance, and superseded conference reports were excluded. Because this was not a systematic review, selective evidence identification remains a potential limitation.

### 2.2. Evidence Integration and Development of the Framework

We grouped the information used in the framework into three categories. “Direct drug-specific evidence” denotes findings from pivotal trials or current product labels. “Indirect clinical inference” denotes conclusions based on the known toxicity profile, biological plausibility, trial eligibility criteria, or extrapolation from related ADCs without direct comparative validation. “Practical considerations” include treatment logistics, specialist access, patient preferences, and healthcare system resources. We did not assign numerical weights or evidence scores, and these factors are not intended to rank one anti-TROP2 ADC above the other.

### 2.3. PTOK Endorsement Process

The framework was developed as clinical guidance within the Polish Society of Clinical Oncology (PTOK). The participating authors include members of the PTOK Breast Cancer Section and the Society’s President and Vice-President. The authors reviewed the manuscript and recommendations, agreed by consensus, and the PTOK Breast Cancer Section formally approved them in September 2026. This endorsement reflects the Society’s expert position but is distinct from prospective clinical validation, which has not yet been performed. P.J.W. and E.W. conducted the evidence review and data curation, with the participating authors providing clinical interpretation and verification. Conflicts of interest are disclosed and were considered during review.

## 3. Regulatory Approval, Trial Populations, and Maturity of Clinical Data

Regulatory indications, pivotal-trial populations, and guideline positioning should be considered separately, as they are not interchangeable (Table 1). Survival evidence also differs by clinical setting. In previously treated triple-negative breast cancer (TNBC), SG improved overall survival (OS) in ASCENT. In HR-positive/HER2-negative (HR+/HER2−) advanced breast cancer after chemotherapy, SG improved OS in TROPiCS-02, whereas Dato-DXd improved progression-free survival (PFS) but not OS in TROPION-Breast01 [7,8,9]. In first-line advanced TNBC not eligible for PD-1/PD-L1 inhibitor therapy, Dato-DXd improved OS in TROPION-Breast02, while OS data from ASCENT-03 were immature at the primary analysis [10,11]. ASCENT-07, conducted in an earlier-line HR+/HER2− setting, did not meet its primary PFS endpoint and had immature OS data [12]. These are setting-specific results and should not be interpreted as evidence of a difference between SG and Dato-DXd.

## 4. Mechanistic Basis of the Clinical Differences

Both agents target TROP2 but differ substantially in molecular design. SG combines a humanized anti-TROP2 antibody with SN-38, the active metabolite of irinotecan, through a hydrolyzable CL2A linker and has a drug-to-antibody ratio (DAR) of approximately 7.6. Dato-DXd uses a more plasma-stable tetrapeptide linker (GGFG) and the exatecan-derived topoisomerase I inhibitor DXd, with a lower DAR of approximately 4 [15].

DXd is a highly potent, membrane-permeable topoisomerase I inhibitor that can produce a bystander effect after internalization and lysosomal processing [15,16]. Payload, linker stability, DAR, and pharmacokinetic characteristics provide a biologically plausible basis for differences in activity and toxicity. However, the clinical contribution of any individual design feature cannot be isolated from the available non-comparative data, and mechanistic differences should not be used as evidence of comparative efficacy.

Interstitial lung disease (ILD)/pneumonitis is not a characteristic toxicity signal of SG but is an established risk with Dato-DXd. The distinct linker–payload platforms provide a plausible mechanistic explanation, but this remains an inference rather than a clinically validated causal comparison. Risk factors derived from trastuzumab deruxtecan (T-DXd) should therefore be clearly distinguished from risk factors documented specifically with Dato-DXd.

## 5. Current Positioning in International Guidelines

International guidelines position SG and Dato-DXd according to disease subtype, treatment history, biomarker context, and jurisdiction. These recommendations should be reported as written and should not be treated as a direct ranking between the drugs. Moreover, ESMO evidence/recommendation grades, ESMO Magnitude of Clinical Benefit Scale (MCBS) scores, NCCN categories and preferred-status designations, AGO levels of evidence/grades of recommendation, and ASCO recommendations are different systems and are not directly interchangeable (Table 2).

In HR+/HER2− disease after chemotherapy, TROPiCS-02 reported an OS benefit with SG (14.4 vs. 11.2 months; HR 0.79; 95% CI 0.65–0.96), whereas TROPION-Breast01 did not show a statistically significant OS difference with Dato-DXd (18.6 vs. 18.3 months; HR 1.01; 95% CI 0.83–1.22) [8,9]. Because these results come from separate trials, they should not be read as evidence of a survival difference between SG and Dato-DXd. Likewise, the rationale for individual guideline recommendations should not be inferred unless it is explicitly stated.

This positioning may evolve as new evidence becomes available. In first-line advanced TNBC not eligible for PD-1/PD-L1 inhibitor therapy, both SG and Dato-DXd have received NCCN category 1 recommendations. At present, a significant OS benefit has been demonstrated only for Dato-DXd in TROPION-Breast02, whereas OS data from ASCENT-03 remain immature.

## 6. Limitations of Indirect Cross-Trial Comparisons

No randomized head-to-head trial has compared SG with Dato-DXd, and cross-trial differences in efficacy or safety cannot establish their relative treatment effect. Differences in patient populations, prior therapy, comparator use, response assessment, follow-up, subsequent treatment, exposure, and adverse-event reporting limit comparability (Table 3). The side-by-side tables are therefore descriptive only; within-trial differences versus control should not be interpreted as indirect comparisons between the two ADCs. A formal anchored indirect comparison would require a sufficiently comparable common comparator, which the available datasets do not support.

## 7. Are Current Data Sufficient for Optimal Patient Selection?

When both agents are appropriate options, selection should first be based on regulatory and guideline eligibility, then on safety, patient vulnerabilities, prior ADC exposure, monitoring requirements, treatment burden, and preferences. TROP2 expression is not a validated predictive biomarker for choosing SG versus Dato-DXd, and no other biomarker has been prospectively validated for this purpose. These practical considerations should be adapted to local access, reimbursement, monitoring infrastructure, and specialist availability.

## 8. Activity Profile—Probability and Timing of Response

### 8.1. HR+/HER2− After Chemotherapy

Table 4 summarizes the key efficacy results for SG and Dato-DXd in HR+/HER2− disease after chemotherapy.

### 8.2. TNBC—First Line (PD-L1/PD-1-Ineligible)

The two first-line TNBC trials showed different within-trial response patterns (Table 5): ASCENT-03 reported a small absolute ORR difference between SG and its chemotherapy control despite PFS and DOR benefits, whereas TROPION-Breast02 reported a larger ORR difference between Dato-DXd and its own control [10,11]. Because the control-arm ORRs and other trial characteristics differed, these observations should remain descriptive and hypothesis-generating. They do not demonstrate that Dato-DXd has a higher response probability, greater depth of response, or greater ability to reverse organ dysfunction than SG.

Reliable comparative evidence regarding response onset is unavailable. Median time to response was 1.6 months in ASCENT-03, while the single-arm TROPION-PanTumor02 TNBC cohort reported a median of 1.4 months [21]. These data arise from different studies and populations and should not be interpreted as demonstrating equivalent or different response kinetics. Neither drug has been prospectively evaluated specifically in a visceral- or organ-crisis population.

Therefore, current data do not support choosing either SG or Dato-DXd based on response probability or speed alone. When substantial or rapid tumor shrinkage is clinically important, treatment choice should instead consider eligibility, the available trial evidence, safety, organ function, and individual patient factors.

## 9. Safety Profile

### 9.1. Overall Toxicity—Two Distinct Clinical Signatures

Despite sharing the same target, SG and Dato-DXd have distinct toxicity profiles. SG is characterized mainly by hematologic and gastrointestinal toxicity, whereas Dato-DXd is more commonly associated with oral mucositis/stomatitis, ocular toxicity, and ILD/pneumonitis (Table 6). However, toxicity percentages from different trials are not directly comparable because populations, treatment exposure, grading versions, event grouping, attribution (treatment-emergent versus treatment-related), and reporting thresholds differ. Where possible, this review reports the event definition used by the source and avoids imputing zero incidence when an event was simply not reported. TROPION-Breast01 graded adverse events using CTCAE version 5.0; ASCENT used its protocol-specified CTCAE framework. Because versions, event grouping, attribution and reporting thresholds were not fully harmonized, numerical cross-trial toxicity comparisons remain inappropriate.

### 9.2. Severe and Potentially Life-Threatening Complications

Treatment-related deaths were uncommon and are not directly comparable across trials (Table 7). In ASCENT-03, fatal events were mainly infections associated with neutropenia. Current U.S. prescribing information for SG recommends primary G-CSF prophylaxis from cycle 1 in patients at increased risk of febrile neutropenia, with blood-count monitoring and treatment modification according to neutrophil thresholds [23]. Severe diarrhea requires prompt supportive care and dose interruption or reduction.

Dato-DXd is associated with a risk of severe or fatal ILD/pneumonitis. In the pooled breast cancer safety population, ILD/pneumonitis occurred in 3.0% of patients, including 0.4% grade 3 and 0.2% fatal events [13]. Renal impairment was associated with a higher incidence, whereas other proposed risk factors are derived mainly from T-DXd experience and have not been validated specifically for Dato-DXd [24]. Suspected ILD/pneumonitis requires treatment interruption and evaluation; confirmed symptomatic grade ≥2 events require permanent discontinuation and systemic corticosteroids [13].

### 9.3. Practical Monitoring and Supportive Care

Dato-DXd requires structured ocular and oral supportive care. Current U.S. prescribing information recommends ophthalmic assessment at baseline and end of treatment, with visual acuity and slit-lamp examination every three cycles, regular preservative-free lubricating eye drops, and prompt evaluation of new ocular symptoms [13]. Stomatitis prophylaxis includes steroid-containing mouthwash from treatment initiation and oral cryotherapy during infusion, with dose modification according to severity [13]. Thus, the once-every-three-week schedule does not necessarily reduce overall treatment burden.

## 10. Evidence-Informed Framework for Treatment Selection

When both agents are appropriate, select sequentially: confirm regulatory and guideline eligibility, assess major safety concerns and modifiable risks, consider prior ADC exposure, and incorporate patient preferences and treatment burden. Conflicting factors require individualized clinical judgment rather than a scoring approach. Table 8 separates direct drug-specific evidence, indirect clinical inference, and practical considerations; these categories indicate the source of the rationale, not comparative evidence strength. The framework is most applicable to ADC-naive patients, and no validated sequencing rule exists for SG versus Dato-DXd after prior topoisomerase I ADC exposure. Comparative response probability and kinetics remain uncertain; therefore, a need for rapid or substantial tumor shrinkage does not currently favor either agent.

UGT1A1*28/*28 is associated with increased SG-related toxicity, particularly severe neutropenia and diarrhea [25]. UGT1A1 status is not a validated biomarker for choosing between SG and Dato-DXd, and routine pretreatment genotyping is not required. When available, genotype information may guide closer toxicity monitoring but should not determine treatment selection.

## 11. ADC Sequencing and Future Directions

### 11.1. Prior ADC Exposure, Sequencing, and Potential Cross-Resistance

Prior ADC exposure is clinically relevant to the framework’s applicability, but available sequencing evidence is indirect. Retrospective cohorts evaluating sequential SG and T-DXd suggest shorter PFS with the second topoisomerase I inhibitor–based ADC [26,27]. These studies do not directly compare SG with Dato-DXd and cannot separate pharmacologic cross-resistance from the general loss of efficacy expected in later treatment lines.

Additional hypothesis-generating evidence comes from TROPION-PanTumor01 [28], in which the ORR with Dato-DXd was 52% among patients with TNBC who were naive to prior topoisomerase I inhibitor–based ADC therapy, compared with 34% in the overall TNBC cohort. Because these populations overlap, this comparison cannot quantify the effect of prior ADC exposure. Nevertheless, the finding is directionally consistent with reduced activity after prior topoisomerase I ADC exposure.

A biological basis for such cross-resistance is increasingly plausible. Preclinical models suggest that resistance to topoisomerase I ADCs may be driven by payload-related mechanisms, including drug-efflux pathways, and that switching to a mechanistically different payload can restore antitumor activity [29]. Clinical genomic data have also identified acquired TOP1 mutations associated with resistance to both SN-38 and deruxtecan payloads [30]. These findings support the biological plausibility of cross-resistance but do not establish its magnitude in patients.

Prospective studies are needed to define optimal ADC sequencing. STORM and ALTER evaluate strategies involving SG and T-DXd [31,32]; they may inform broader principles of ADC sequencing but will not directly resolve every aspect of SG-versus-Dato-DXd selection. Dedicated prospective evidence will be required for this specific question.

### 11.2. Personalization Based on the HER2 Expression Gradient—A Future Direction

Although outside the direct SG-versus-Dato-DXd comparison, HER2 expression may influence whether T-DXd is used before an anti-TROP2 ADC and therefore indirectly affect treatment sequencing. DESTINY-Breast04 and DESTINY-Breast06 established substantial activity of T-DXd across HER2-low and, more recently, HER2-ultralow disease [33,34], but whether HER2 expression within this spectrum should guide ADC selection remains uncertain [35].

A large real-world analysis of 4030 patients generated a hypothesis regarding HER2-expression gradients and ADC sequencing. Importantly, the reported endpoint was time on treatment rather than progression-free survival and is susceptible to treatment-selection bias, treatment discontinuation for non-progression reasons, and unmeasured confounding. Any subgroup findings should therefore remain exploratory and should not be used as a validated rule for choosing or sequencing anti-TROP2 ADCs [36].

HER2 expression should therefore not currently be interpreted as a validated continuous biomarker for choosing between T-DXd and an anti-TROP2 ADC. Rather, it may be a future component of ADC sequencing strategies, alongside clinical efficacy, toxicity, prior ADC exposure, and patient-specific factors.

## 12. Conclusions

Sacituzumab govitecan and datopotamab deruxtecan target TROP2 but differ in clinical evidence, toxicity, monitoring, and treatment logistics. Without head-to-head trials, these differences do not establish superiority of either agent, and cross-trial efficacy or safety differences cannot substitute for direct comparative evidence.

The framework first establishes treatment eligibility and then considers safety, comorbidities, prior ADC exposure, supportive-care needs, patient priorities, treatment burden, and healthcare system resources. It is PTOK-endorsed guidance rather than a validated treatment selection algorithm and requires prospective evaluation.

## Figures and Tables

**Table 1:** Phase III evidence, pivotal-trial populations, and regulatory context by disease setting. Regulatory indications, trial populations, and guideline positioning are shown as distinct concepts and should not be interpreted as interchangeable.

Disease Setting	SG: Phase III Evidence/Regulatory Context	Dato-DXd: Phase III Evidence/Regulatory Context	Interpretive Caveat
TNBC, previously treated/regulatory context	ASCENT (2020); full FDA approval 2021; OS HR 0.51 (95% CI 0.41–0.62) in the full randomized population; HR 0.48 (95% CI 0.38–0.59) in the primary population without brain metastases [1]	U.S. FDA indication (May 2026): unresectable/metastatic TNBC in adults who are not candidates for PD-1/PD-L1 inhibitor therapy; the U.S. indication itself does not specify a line of therapy. The pivotal TROPION-Breast02 population was untreated for advanced disease [2,11,13].	ASCENT evaluated previously treated TNBC, whereas the current Dato-DXd U.S. indication is broader than the pivotal first-line population. Regulatory scope should not be inferred from trial line alone.
HR+/HER2− (post-chemotherapy)	TROPiCS-02 (2022–2023); OS HR 0.79 (95% CI 0.65–0.96) [9]	TROPION-Breast01 (FDA approval January 2025 [5]; EU marketing authorization April 2025 [6]); final OS HR 1.01 (95% CI 0.83–1.22) [7,8]	Both agents have mature trial-specific OS analyses in this setting; statistical significance across separate trials must not be interpreted as a direct treatment comparison.
TNBC, 1st line, PD-L1/PD-1-ineligible	ASCENT-03 (NEJM 2025); PFS HR 0.62 (95% CI 0.50–0.77); OS immature at first analysis [10]	TROPION-Breast02 (full publication 2026); PFS HR 0.57 (99% CI 0.44–0.73) and OS HR 0.79 (95.01% CI 0.64–0.98) [11]	Both trials studied untreated advanced TNBC patients ineligible for PD-1/PD-L1 therapy, but comparator composition, populations, and follow-up differ; no head-to-head inference is valid.
TNBC, 1st line, PD-L1-positive	ASCENT-04/KEYNOTE-D19 (SG + pembrolizumab; NEJM 2026; OS promising but immature) [14]	No analogous indication	A unique niche for SG in combination with immunotherapy

Abbreviations: FDA, U.S. Food and Drug Administration; HR, hazard ratio; HR+/HER2−, hormone receptor-positive/HER2-negative; OS, overall survival; PD-1/PD-L1, programmed cell death protein 1/programmed death-ligand 1; PFS, progression-free survival; TNBC, triple-negative breast cancer.

**Table 2:** Selected international guideline positioning of SG and Dato-DXd across relevant breast cancer subtypes and clinical settings. Guideline systems use different grading frameworks; categories and scores should be interpreted within each guideline and not compared across organizations.

Breast Cancer Subtype/Clinical Setting	Guideline	Sacituzumab Govitecan	Datopotamab Deruxtecan
HR+/HER2− advanced BC after endocrine therapy and chemotherapy	ESMO Living Guideline (MCBS v2.0) [17]	I, B; MCBS 4	I, B; MCBS 3
HR+/HER2− advanced BC after endocrine therapy and chemotherapy	NCCN v4.2026 [18]	Category 1, preferred second-line option	Other recommended regimen (Category 2A), second-line or subsequent therapy after prior endocrine-based therapy and chemotherapy for unresectable/metastatic HR+/HER2− disease; NCCN v4.2026, BINV-Q systemic-therapy pathway.
HR+/HER2− advanced BC after endocrine therapy and chemotherapy	AGO (Germany) 2026.1 [19]	LoE 1b, GR A (++)	LoE 1b, GR B (+)
HR+/HER2− advanced BC after endocrine therapy and chemotherapy	ASCO Rapid Recommendation Update (2023) [20]	May be offered after endocrine therapy and ≥2 prior chemotherapy lines for metastatic HR+/HER2− disease	No dedicated ASCO rapid recommendation update specifically incorporating Dato-DXd full approval was identified by the evidence cutoff; absence of a rapid update should not be interpreted as a negative recommendation.
First-line advanced TNBC not eligible for PD-1/PD-L1 inhibitor therapy	NCCN v4.2026 [18]	Category 1	Category 1

Abbreviations: AGO, Arbeitsgemeinschaft Gynäkologische Onkologie; ASCO, American Society of Clinical Oncology; ESMO, European Society for Medical Oncology; GR, grade of recommendation; HR+/HER2−, hormone receptor-positive/HER2-negative; LoE, level of evidence; MCBS, Magnitude of Clinical Benefit Scale; NCCN, National Comprehensive Cancer Network.

**Table 3:** Key characteristics of the four phase III trials most relevant to indirect SG–Dato-DXd interpretation. The table is descriptive and highlights sources of non-exchangeability between studies.

Trial/Setting	Prior-Treatment Requirements	Chemotherapy Comparator	Response Assessment	Follow-Up/Subsequent Therapy Context
TROPiCS-02 [9]—HR+/HER2− after chemotherapy	Prior endocrine therapy, taxane and CDK4/6 inhibitor; 2–4 prior chemotherapy regimens for metastatic disease	Eribulin, vinorelbine, capecitabine, or gemcitabine	BICR, RECIST v1.1	Median follow-up 12.5 months at the protocol-specified OS analysis; later-line, heavily pretreated population.
TROPION-Breast01 [7,8]—HR+/HER2− after chemotherapy	Progression on endocrine therapy; 1–2 prior chemotherapy lines in the inoperable/metastatic setting	Eribulin, vinorelbine, capecitabine, or gemcitabine	BICR, RECIST v1.1	Median follow-up 22.8 months at final OS analysis; subsequent ADC use 12.3% with Dato-DXd vs. 24.0% with chemotherapy.
ASCENT-03 [10]—untreated advanced TNBC, PD-1/PD-L1 inhibitor not appropriate	No prior systemic therapy for advanced disease; patients not candidates for PD-1/PD-L1 inhibitor therapy	Paclitaxel, nab-paclitaxel, or gemcitabine/carboplatin	BICR, RECIST v1.1	OS immature at primary analysis; protocol permitted on-study SG after BICR-confirmed progression in eligible control patients.
TROPION-Breast02 [11]—untreated advanced TNBC, immunotherapy not an option	No prior systemic therapy for advanced disease; immunotherapy not an option	Paclitaxel/nab-paclitaxel, capecitabine, carboplatin, or eribulin	BICR, RECIST v1.1	Median study follow-up 27.5 months at the August 2025 data cutoff reported in updated analyses.

Abbreviations: ADC, antibody–drug conjugate; BICR, blinded independent central review; CDK4/6, cyclin-dependent kinase 4/6; HR+/HER2−, hormone receptor-positive/HER2-negative; OS, overall survival; PD-1/PD-L1, programmed cell death protein 1/programmed death-ligand 1; RECIST, Response Evaluation Criteria in Solid Tumors; TNBC, triple-negative breast cancer.

**Table 4:** Trial-specific clinical activity in HR+/HER2− disease. Results are presented for separate randomized trials and must not be interpreted as a head-to-head comparison.

Parameter	SG—TROPiCS-02	Dato-DXd—TROPION-Breast01
ORR (ADC vs. ChT)	21% vs. 14%	36.4% vs. 22.9%
Δ ORR (pp) *	+7	+13.5
PD as BOR (ADC vs. ChT)	21% vs. 28%	15.9% vs. 20.7%
Δ PD (pp) *	−7	−4.8
Median PFS	5.5 vs. 4.0 mo (HR 0.66; 95% CI 0.53–0.83)	6.9 vs. 4.9 mo (HR 0.63; 95% CI 0.52–0.76)
Median OS	14.4 vs. 11.2 mo (HR 0.79; 95% CI 0.65–0.96; *p* = 0.02)	18.6 vs. 18.3 mo (HR 1.01; 95% CI 0.83–1.22; *p* = 0.9445)
DOR	8.1 vs. 5.6 mo	6.7 vs. 5.7 mo

Abbreviations: ADC, antibody–drug conjugate; BOR, best overall response; ChT, chemotherapy; DOR, duration of response; HR, hazard ratio; ORR, objective response rate; OS, overall survival; PD, progressive disease; PFS, progression-free survival; pp, percentage points. TROPiCS-02 and TROPION-Breast01 used the same menu of physician’s-choice chemotherapy options (eribulin, vinorelbine, capecitabine, gemcitabine), but their populations, prior-treatment distributions, follow-up, subsequent therapy, and other trial characteristics differ. *Δ rows show each ADC’s within-trial difference versus its own control and are descriptive only; they are not an indirect SG-versus-Dato-DXd estimate. Efficacy endpoints were reported at prespecified analyses and may derive from different data cutoffs; values are shown as reported in the cited publications.

**Table 5:** Trial-specific clinical activity in first-line TNBC in patients not eligible for PD-1/PD-L1 inhibitor therapy. Results derive from separate trials and must not be interpreted as a head-to-head comparison.

Parameter	SG—ASCENT-03 [10]	Dato-DXd—TROPION-Breast02 [11]
ORR (ADC vs. ChT)	48% vs. 46%	63% vs. 29%
Δ ORR (pp) *	+2	+34
PD as BOR (ADC vs. ChT)	5% vs. 13%	8% vs. 16%
Δ PD (pp) *	−8	−8
DOR	12.2 vs. 7.2 mo	12.3 vs. 7.1 mo
Median PFS	9.7 vs. 6.9 mo (HR 0.62; 95% CI 0.50–0.77)	10.8 vs. 5.6 mo (HR 0.57; 99% CI 0.44–0.73)
OS	immature at first analysis	23.7 vs. 18.7 mo (HR 0.79; 95.01% CI 0.64–0.98; *p* = 0.029)

Abbreviations: ADC, antibody–drug conjugate; BOR, best overall response; ChT, chemotherapy; DOR, duration of response; HR, hazard ratio; ORR, objective response rate; OS, overall survival; PD, progressive disease; PD-1/PD-L1, programmed cell death protein 1/programmed death-ligand 1; PFS, progression-free survival; pp, percentage points; TNBC, triple-negative breast cancer. Comparator composition and other trial characteristics differ between ASCENT-03 and TROPION-Breast02 (see Section 6). *Δ rows show each ADC’s within-trial difference versus its own control and are descriptive only; they do not estimate a comparative SG-versus-Dato-DXd treatment effect.

**Table 6:** Selected safety findings from ASCENT (data cutoff, 11 March 2020) and the primary TROPION-Breast01 analysis (data cutoff, 17 July 2023), with pooled-label data for Dato-DXd ILD/pneumonitis from the May 2026 U.S. prescribing information where indicated. The source populations and safety-reporting conventions differ; percentages are descriptive and should not be interpreted as comparative toxicity rates.

Treatment-Related Adverse Event	SG ASCENT TNBC ≥ 2L, Safety n = 258 Any Grade	SG ASCENT TNBC ≥ 2L, Safety n = 258 Grade ≥ 3	Dato-DXd TROPION-Breast01 HR+/HER2−, Safety n = 360 Any Grade	Dato-DXd TROPION-Breast01 HR+/HER2−, Safety n = 360 Grade ≥ 3/4
Neutropenia *	63%	51%	10.8%	1.1%
Diarrhea	59%	10%	7.5%	NR
Nausea	57%	~3%	51.1%	1.4%
Stomatitis	NR	NR	50.0%	6.4%
Alopecia	46%	NR	36.4%	0% reported in source table
Fatigue	NR	NR	23.6%	1.7%
Dry eye	<10% †	0% †	21.7%	0.6%
Vomiting	NR	NR	19.7%	1.1%
Febrile neutropenia	6%	6%	NR	NR
Keratitis/corneal events	No characteristic signal identified	NR	Keratitis 14.4% †	0.6% †
ILD/pneumonitis	No characteristic signal identified	NR	<10% in trial AE table; 3.0% in pooled breast-cancer safety population ‡	0.4% grade 3 in pooled breast-cancer safety population ‡

Abbreviations: AE, adverse event; HR+/HER2−, hormone receptor-positive/HER2-negative; ILD, interstitial lung disease; NR, not reported; TRAE, treatment-related adverse event; TNBC, triple-negative breast cancer. Note. ASCENT and TROPION-Breast01 enrolled different populations and used different safety-reporting conventions; values are therefore trial-specific and non-comparative. “NR” means not reported in the selected source table and must not be interpreted as zero incidence. Exposure also differed across studies and should be considered when interpreting cumulative toxicities. * For Dato-DXd, neutropenia was reported using grouped terms in the cited safety analysis. † In TROPION-Breast01, keratitis was reported as a grouped term comprising keratitis, punctate keratitis, and ulcerative keratitis. ‡ Current U.S. labeling reports ILD/pneumonitis in a pooled breast cancer safety population; the pooled estimate is provided to characterize this clinically important toxicity and is not a trial-matched comparison with SG [13]. Selected trial tables apply reporting thresholds. Absence from such a table does not establish a 0% incidence; events not quantified in the relevant source are therefore shown as “NR”. Treatment modification rates provide additional context but remain trial-specific. In ASCENT, adverse events led to SG dose reduction in 22% of patients, treatment interruption in approximately 61%, and treatment discontinuation in approximately 5% [1]. In TROPION-Breast01, treatment-related adverse events led to Dato-DXd dose reduction in 20.8%, interruption in 11.9%, and discontinuation in 2.5% at the primary PFS analysis; updated final-OS reporting showed broadly similar patterns, with dose reduction in 24.2% and discontinuation in 3.3% [7,8,22]. These figures should not be interpreted as comparative estimates because treatment exposure, attribution rules, and toxicity management protocols differed across trials.

**Table 7:** Treatment-related deaths reported in pivotal trials. Denominators are shown for each ADC-treated safety population where available; counts are descriptive and should not be interpreted as comparative fatal toxicity rates.

Trial	Agent	Treatment-Related Deaths	Cause
ASCENT (TNBC ≥ 2L)	SG	0/258	—
TROPiCS-02 (HR+/HER2−)	SG	1/268 (0.4%)	Septic shock/neutropenic enterocolitis
ASCENT-03 (1L TNBC)	SG	6/275 (2.2%)	All six treatment-related deaths were infections; five occurred with neutropenia in patients with febrile-neutropenia risk factors who had not received primary G-CSF prophylaxis.
TROPION-Breast01 (HR+/HER2−)	Dato-DXd	1/365 (0.3%)	ILD/pneumonitis
TROPION-Breast02 (1L TNBC)	Dato-DXd	0 (1*)/319	—

* FDA label: one fatal adverse reaction due to ILD/pneumonitis was reported in TROPION-Breast02. Abbreviations: G-CSF, granulocyte colony-stimulating factor; HR+/HER2−, hormone receptor-positive/HER2-negative; ILD, interstitial lung disease; TNBC, triple-negative breast cancer. “—“ means no data.

**Table 8:** Factors that may inform individualization of anti-TROP2 ADC choice based on patient clinical profile.

Clinical Consideration	Relevance to SG	Relevance to Dato-DXd	Evidence Basis
Renal impairment (CrCl 30–<90 mL/min) *	May support consideration of SG	Higher incidence of ILD/pneumonitis has been reported in patients with renal impairment; closer monitoring is recommended	Drug-specific label evidence; treatment-selection implication remains unvalidated
Pre-existing pulmonary disease, previous ILD, or prior thoracic radiotherapy	No characteristic ILD signal	May increase concern regarding ILD/pneumonitis	Indirect clinical inference, partly extrapolated from experience with other DXd-based ADCs
Clinically relevant corneal or ocular disease	May support consideration of SG	Requires careful baseline assessment and ophthalmic monitoring; patients with clinically significant corneal disease were excluded from pivotal trials	Indirect clinical inference based on trial eligibility and the established ocular toxicity profile
Limited bone-marrow reserve or high risk of febrile neutropenia	Greater concern because severe neutropenia is common; current U.S. label recommends cycle-1 primary G-CSF prophylaxis for patients at increased febrile-neutropenia risk.	Severe neutropenia was uncommon in TROPION-Breast01	Drug-specific toxicity data; the treatment-selection implication is indirect
Chronic diarrhea or inflammatory bowel disease	Greater concern because diarrhea is a characteristic toxicity	May support consideration of Dato-DXd	Indirect clinical inference based on differential toxicity profiles
History of severe or recurrent stomatitis or clinically relevant oral disease	May support consideration of SG	Greater concern because stomatitis is a frequent and clinically relevant toxicity	Indirect clinical inference based on differential toxicity profiles
UGT1A1 *28/*28 genotype	Associated with an increased risk of severe neutropenia and diarrhea; warrants closer monitoring and may influence treatment choice	No corresponding pharmacogenetic concern has been established	Drug-specific pharmacogenetic evidence; not a validated indication to prefer Dato-DXd
Limited access to ophthalmology or rapid evaluation of suspected ILD	No additional specialist monitoring specific to these toxicities	May make safe monitoring more difficult	Practical healthcare system consideration
Preference for fewer infusion visits	Administered on days 1 and 8 of each 21-day cycle	Administered once every 3 weeks	Practical consideration based on dosing schedules
Priority placed on treatment supported by a demonstrated OS benefit in the relevant clinical setting	Demonstrated OS benefit in HR+/HER2− disease after chemotherapy and in previously treated TNBC; OS immature in first-line TNBC at the primary ASCENT-03 analysis	Demonstrated OS benefit in first-line TNBC in patients not eligible for PD-1/PD-L1 inhibitors; no significant OS benefit in TROPION-Breast01	Direct trial evidence; results are setting-specific and do not establish comparative superiority
Need for a high probability of substantial tumor shrinkage	No validated comparative evidence establishes a higher probability or depth of response with SG.	No validated comparative evidence establishes a higher probability or depth of response with Dato-DXd.	Insufficient comparative evidence; within-trial ORR differences across separate studies are descriptive only.
Need for rapid onset of response	Available data do not demonstrate a faster onset of response with SG.	Available data do not demonstrate a faster onset of response with Dato-DXd.	Insufficient comparative evidence; cross-trial TTR values cannot establish equivalent or different response kinetics.
Regulatory/guideline eligibility for the specific setting	Confirm indication and guideline-supported use before considering preference factors.	Confirm indication and guideline-supported use before considering preference factors.	Required first step; jurisdiction- and setting-specific.
Prior topoisomerase I ADC exposure	Activity of a subsequent ADC may be reduced; no validated SG-versus-Dato-DXd sequencing rule.	Activity of a subsequent ADC may be reduced; no validated SG-versus-Dato-DXd sequencing rule.	Retrospective/hypothesis-generating evidence; confounded by later treatment line.
Overall treatment burden and patient preference	Day 1 and Day 8 infusions every 21 days; hematologic/GI monitoring and supportive care may add burden.	Once every 3 weeks, but scheduled ophthalmic monitoring, eye care, stomatitis prophylaxis, and ILD vigilance add burden.	Practical consideration; fewer infusions do not necessarily mean lower total treatment burden.

Abbreviations: ADC, antibody–drug conjugate; CrCl, creatinine clearance; GI, gastrointestinal; HR+/HER2−, hormone receptor-positive/HER2-negative; ILD, interstitial lung disease; OS, overall survival; PD-1/PD-L1, programmed cell death protein 1/programmed death-ligand 1; TTR, time to response; UGT1A1, uridine diphosphate glucuronosyltransferase 1A1. * Dato-DXd: no dose adjustment is required for CrCl 30–<90 mL/min; however, current prescribing information recommends monitoring for increased adverse reactions, including ILD/pneumonitis, in renal impairment. SG: no dose adjustment is required for mild-to-moderate renal impairment, with no analogous ILD-specific monitoring requirement. These pharmacokinetic/label observations do not constitute validated comparative treatment selection criteria. Patient-reported outcomes should be interpreted within individual trials, not across them. Dato-DXd delayed deterioration in global health status/quality of life, pain, and physical functioning versus chemotherapy in TROPION-Breast01 [22]. In TROPiCS-02, SG delayed deterioration in global health status/quality of life and fatigue, but not pain [9]. These findings are trial-specific and do not establish a quality-of-life advantage for either ADC.

## Data Availability

No new data were created or analyzed in this study. Data sharing is not applicable to this article.

## Abbreviations

The following abbreviations are used in this manuscript:

ADC	antibody–drug conjugate
AGO	Arbeitsgemeinschaft Gynäkologische Onkologie
ASCO	American Society of Clinical Oncology
BOR	best overall response
CHMP	Committee for Medicinal Products for Human Use
ChT	chemotherapy
CrCl	creatinine clearance
DAR	drug-to-antibody ratio
Dato-DXd	datopotamab deruxtecan
DCR	disease control rate
DOR	duration of response
DXd	deruxtecan payload
ESMO	European Society for Medical Oncology
EU	European Union
FDA	U.S. Food and Drug Administration
G-CSF	granulocyte colony-stimulating factor
GR	grade of recommendation
HER2	human epidermal growth factor receptor 2
HR	hormone receptor; hazard ratio
HR+/HER2−	hormone receptor-positive/HER2-negative
ILD	interstitial lung disease
LoE	level of evidence
MCBS	Magnitude of Clinical Benefit Scale
NCCN	National Comprehensive Cancer Network
ORR	objective response rate
OS	overall survival
PD	progressive disease
PD-1	programmed cell death protein 1
PD-L1	programmed death-ligand 1
PFS	progression-free survival
pp	percentage points
SG	sacituzumab govitecan
SN-38	active metabolite of irinotecan
T-DXd	trastuzumab deruxtecan
TNBC	triple-negative breast cancer
TOP1	topoisomerase I
TROP2	trophoblast cell-surface antigen 2
TTR	time to response
UGT1A1	uridine diphosphate glucuronosyltransferase 1A1
